# Experienced Burden of and Adherence to Smartphone-Based Ecological Momentary Assessment in Persons with Affective Disorders

**DOI:** 10.3390/jcm9020322

**Published:** 2020-01-23

**Authors:** Claire R. van Genugten, Josien Schuurmans, Femke Lamers, Harriëtte Riese, Brenda W. J. H. Penninx, Robert A. Schoevers, Heleen M. Riper, Johannes H. Smit

**Affiliations:** 1 Department of Research and Innovation, GGZ inGeest, Specialized Mental Health Care, 1081 Amsterdam, The Netherlands; j.schuurmans@ggzingeest.nl (J.S.); b.penninx@amsterdamumc.nl (B.W.J.H.P.); h.riper@vu.nl (H.M.R.); jh.smit@ggzingeest.nl (J.H.S.); 2 Department of Psychiatry, Amsterdam Public Health Research Institute, Amsterdam UMC, Vrije Universiteit, 1081 Amsterdam, The Netherlands; f.lamers@ggzingeest.nl; 3 Interdisciplinary Centre for Psychopathology and Emotion Regulation, Department of Psychiatry, University Medical Centre Groningen, University of Groningen, 9713 Groningen, The Netherlands; h.riese@umcg.nl (H.R.); r.a.schoevers@umcg.nl (R.A.S.); 4 Department of Clinical, Neuro and Developmental Psychology, Clinical Psychology Section, Vrije Universiteit Amsterdam and the Amsterdam Public Health Research Institute, 1081 Amsterdam, The Netherlands; 5 Institute of Telepsychiatry, University of Southern Denmark, 5000 Odense, Denmark

**Keywords:** affective disorders, depression, anxiety disorders, ecological momentary assessment, burden, adherence

## Abstract

(1) Background: The use of smartphone-based ecological momentary assessment (EMA) questionnaires in affective disorder research has rapidly increased. Though, a thorough understanding of experienced burden of and adherence to EMA is crucial in determining the usefulness of EMA. (2) Methods: Persons with current affective disorders (*n* = 100), remitted persons (*n* = 190), and healthy controls (*n* = 94) participated in a smartphone-based EMA two-week monitoring period. Our primary outcomes were (momentary) perceived burden of and adherence to EMA. (3) Results: In the whole sample, lower positive and higher negative affect were associated with slightly higher levels of perceived momentary burden (B = −0.23 [95%CI = −0.27–0.19], B = 0.30 [95%CI = 0.24–0.37], respectively). The persons with current affective disorders reported slightly higher levels of experienced momentary burden (Mdn = 1.98 [IQR = 1.28–2.57]), than the remitted persons (Mdn = 1.64 [IQR = 1.11–2.24]) and healthy controls (Mdn = 1.28 [IQR = 1.04–1.92]). Nevertheless, the persons with current affective disorders still showed very high adherence rates (Mdn = 94.3% [IQR = 87.9–97.1]), at rates on a par with the remitted persons (Mdn = 94.3% [IQR = 90.0–97.1]) and healthy controls (Mdn = 94.3% [IQR = 90.0–98.6]). (4) Discussion: Frequent momentary questionnaires of mental well-being are slightly more burdensome to the persons with current affective disorders, but this does not seem to have a negative impact on adherence. Their high rate of adherence to EMA—which was similar to that in remitted persons and healthy controls —suggests that it is feasible to apply (short-duration) EMA.

## 1. Background

In recent decades, we have witnessed a surge in research acknowledging the importance of real-life context and diurnal variation of affective states in persons with affective disorders [1,2,3,4]. Ecological momentary assessment (EMA) is a valuable addition to the traditional methods of studying these dynamics [5,6,7,8]. EMA questionnaires were initially administered via paper-and-pencil diaries. Nowadays, online tools or apps on devices such as mobile telephones are designed to capture momentary ratings. With EMA, participants are asked to self-report information on their momentary affect throughout the day in natural settings [7,8,9], rather than recalling and summarizing their affect within a certain time interval (e.g., last week/month), as is done in retrospective questionnaires [9].

Advocates of EMA argue that assessing affective states more frequently is a more appropriate way to measure affect dynamics, as retrospective distortions are minimized [6,10,11,12,13]. EMA questionnaires provide us with the essential information that needs to be measured in order to generate insights in the temporal variability of anxiety and mood symptoms, the importance of real-life context, phenomenology, and the interrelatedness of symptoms [14,15,16]. These nuances are hard to capture when using traditional retrospective questionnaires, for the reasons mentioned above. 

Measuring affect more frequently means that persons are repeatedly asked to provide information on their own mental well-being. However, since symptoms such as a lack of motivation to act and problems concentrating are core features of the clinical presentation of depression [17,18], adhering to these repeated questionnaires is not self-evident for persons with affective disorders. Also, repeatedly assessing affective states in persons who suffer from a persistently negative mood might lead to high levels of perceived burden. Nevertheless, systematic reviews showed encouraging results regarding the burden experienced and adherence rates by persons with affective disorders [8,14,15]. However, studies only investigated burden in terms of a reflection on the whole monitoring period; momentary burden, e.g., perceived burden at the moment of measuring, was not taken into account in any of these studies. Moreover, the majority of studies reporting on adherence rates used EMA methods such as paper-and-pencil diaries and personal digital assistants (PDAs) instead of smartphone-based EMA questionnaires. Adherence to smartphone-based EMA might differ from other EMA methods; nowadays the use of telephones is incorporated in the daily lives of many persons, as opposed to paper-and-pencil diaries and PDAs that were distributed for study-purposes only. A better understanding of experiences of users is crucial when considering the usefulness of smartphone-based EMA questionnaires for persons with affective disorders.

The aim of this study was to explore perceived burden and adherence to smartphone-based EMA questionnaires in persons with affective disorders sampled from the Ecological Momentary Assessment (EMA) and Actigraphy sub-study (NESDA-EMAA) [19,20]. As well as persons with affective disorders, the cohort also included remitted persons and healthy controls. All (*n* = 384) participated in an interview, including a clinical assessment and an intensive two-week smartphone-based EMA monitoring period with five EMA questionnaires a day. Due to this design, we were able to make a direct comparison in adherence and perceived burden between the persons with current affective disorders and the other two groups in a single cohort.

## 2. Methods

### 2.1. Study Design and Participants

The participants of the Ecological Momentary Assessment (EMA) and Actigraphy sub-study (NESDA-EMAA) were selected from the Netherlands Study of Depression and Anxiety (NESDA). In brief, NESDA is an ongoing longitudinal multi-site naturalistic cohort study which aims to examine the biological, social, and psychological factors contributing to the long-term course of depressive and anxiety disorders [21]. NESDA participants were initially recruited for the baseline measurement between 2004 and 2007 (*n* = 2981) and were invited for a fifth interview on the occasion of the nine-year follow-up measurement (Wave 6; 2014-2017; *n* = 1776). At Wave 6, siblings (*n* = 367) of a subsample of NESDA participants were also recruited and included in the NESDA cohort.

**Figure 1:**
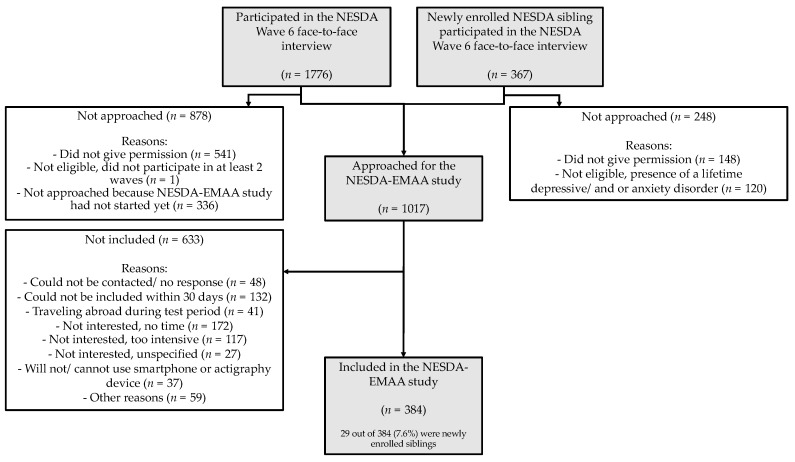
Flowchart of the Ecological Momentary Assessment (EMA) and Actigraphy sub-study (NESDA-EMAA).

Wave 6 comprised a large number of measurements, administered by trained research staff. These included a structured clinical diagnostic interview. After measurement, the participants were invited to join the NESDA-EMAA. For the NESDA-EMAA we invited NESDA participants who participated in at least two of the previous waves, consented to be approached for the NESDA-EMAA, participated in the interview no more than 31 days prior starting with the EMA questionnaires, were familiar with smartphone use and willing to wear a wrist-worn actigraphy device. Siblings were invited if they did not meet the criteria of a current or past diagnosis of depressive and/or anxiety disorder or other severe psychiatric disorder [19,20]. A flowchart showing the enrolment processes is given in Figure 1. 

This resulted in a sample of 384 included participants (of whom 29 were newly enrolled siblings). The sample was divided into three groups: 1) a group with at least one current affective disorder (*n* = 100) (i.e., persons who met the criteria for a depressive or anxiety disorder in the past six months, per to the Diagnostic and Statistical Manual of Mental Disorders, Fourth Edition, Text Revision (DSM-IV-TR) criteria); 2) a group with an affective disorder in remission (*n* = 190) (persons with a life-time diagnosis of depressive and/or anxiety disorder, but who did not meet DSM-IV-TR criteria in the past six months); and 3) a healthy control group (*n* = 94) (persons with no lifetime history of psychiatric disorders). The in- and exclusion criteria are described in more detail elsewhere [19,20]. 

The study was carried out in accordance with the latest version of the Declaration of Helsinki. The Ethical Review Board of VU University Medical Centre Amsterdam and the local review boards of the participating centres provided ethical approval and all participants provided written consent. The NESDA and the NESDA-EMAA are described in more detail elsewhere [19,20,21].

### 2.2. Smartphone-Based Ecological Momentary Assessment Protocol

All the NESDA-EMAA participants were invited to conduct five EMA questionnaires a day for 14 days. These were conducted during the day, using a time-based sampling protocol with fixed time intervals of three hours. The questionnaires were in the form of self-reported answers with a maximum of 31 questions, focusing on momentary affective states, as well as a number of additional items such as hours of sleep since the last questionnaire. Data were collected via smartphones; at an appointed time, the participants received an invitation by text message to conduct and submit the questionnaire. Participants were instructed to complete the questionnaire as soon as possible after receiving the text message, preferably within 15 minutes, but at least within 60 minutes. After completing the questionnaires, the participant’s answers were saved automatically to the secured web-based server (RoQua) [22].

In addition to the automatic reminder, several measures were taken to motivate the participants and to provide support when needed. Research assistants called them on at least two separate occasions: one day and one week after the moment they started with the questionnaires. Additionally, participants received a gift voucher worth €20 and a personalized report of their EMA questionnaires afterwards.

### 2.3. Measurements

#### 2.3.1. Baseline Characteristics 

Basic demographics and clinical characteristics were requested during the Wave 6 measurement. We obtained information about age, gender, and educational level through standard questions. Number of current weekly working hours was assessed by the use of the Treatment Inventory of Costs in Patients with psychiatric disorders (TiC-P) [23]. Presence of lifetime and/or current depressive (dysthymia and major depressive disorder) and anxiety disorders (social anxiety disorder, panic disorder with or without agoraphobia, agoraphobia, and generalized anxiety disorder) was defined according to DSM-IV criteria [24]. Diagnoses were established using the Composite International Diagnostic Interview (CIDI Version 2.1) [25]. This has high validity for the assessment of mental disorders [26].

#### 2.3.2. Momentary Affective States

Momentary affective states were measured repeatedly through the daily EMA questionnaires. The participants provided information about their mental well-being by completing a thirteen-item questionnaire. Twelve of these items were derived from the affect-questionnaire in the Uncovering Positive Potential of Emotional Reactivity (UPPER) study [27]. For this study we added a thirteenth item on feelings of anxiety. The list included seven items covering the negative affective state, whilst the other six covered the positive state. The negative affect items (At this moment I feel upset, irritated, listless/apathic, down, nervous, bored, anxious) were averaged to form a negative affect (NA) subscale and the positive affect ones (At this moment I feel satisfied, relaxed, cheerful, energetic, enthusiastic, calm) were averaged to form a positive affect (PA) subscale. The items were rated on seven-point Likert scales (1 = not at all, 4 = moderate, 7 = very); higher scores meant respectively higher levels of negative and positive affect.

### 2.4. Outcomes

#### 2.4.1. Momentary Burden

Experienced burden as a result of the daily EMA questionnaires was one of the main outcomes of this study. We assessed momentary burden; participants were asked about their experienced burden as a result of the EMA questionnaire itself. They did so by answering the following standard question “How disturbing is filling out a questionnaire right now?”. The question was rated on a seven-point Likert scale (1 = not at all, 4 = moderate, 7 = very).

#### 2.4.2. Experienced Burden Over the Whole Monitoring Period

We also assessed the participants’ overall experience. Immediately after the last daily EMA questionnaire, participants received a text message with a link to an addendum questionnaire, which included four items to evaluate experienced burden over the whole EMA monitoring period. Questions about the study duration; the number of questions; assessment frequency; and overall experience were rated on a seven-point Likert scale (1 = not at all, 4 = moderate, 7 = very). The average of these four items represented the evaluation score (sum possibilities ranging from 1 to 7). The four items showed excellent internal consistency in this sample (Cronbach’s α = 0.924).

#### 2.4.3. Adherence to the Daily EMA Questionnaires

The other main outcome of this study was adherence to the daily EMA questionnaires. This was calculated by counting the percentage of completed EMA questionnaires out of the total of 70 each participant was invited to undertake. Moreover, at the last EMA questionnaire participants were asked to report their main reasons for missing a questionnaire, if they had done so. Possible reasons listed in the questionnaire were: being busy with an activity; no network connection; being asleep; technical problems; not hearing the smartphone; not bringing the smartphone; could not making themselves do it; and “other” reasons. The participants were allowed to select more than one reason. In addition, we counted the absolute number of omissions due to technical issues.

### 2.5. Statistical Analyses

We calculated descriptive statistics for the majority of our variables. Because data appeared to be non-normally distributed based on Kolmogorov–Smirnov tests, we report the median (Mdn) and the interquartile range (IQR) for the continuous variables. Differences between the three diagnosis groups in terms of demographic characteristics, absolute number of uploaded assessments, the retrospective evaluation sum-score, and reported reasons for missing assessments were analyzed; for this we used Kruskall–Wallis tests, Bonferroni-adjusted Mann–Whitney tests, Pearson chi-square tests, and likelihood ratio tests as appropriate.

To analyze the EMA data, we conducted several tests. We looked at whether the groups differed in respect of mean momentary burden as reported through the daily EMA questionnaires. In order to do this, we first calculated the person-mean of this variable by averaging the scores across the participants’ EMA questionnaires. We then compared the averages of the person-mean scores for the three diagnosis groups using a Kruskall–Wallis test and conducted pairwise comparisons using Bonferroni-adjusted Mann–Whitney tests. In addition, we produced a number of generalized estimated equation (GEE) models for the overall sample. We chose GEE models because EMA data have a hierarchical structure; this type of model adjusts for dependency of repeated measures within one participant and can handle non-normally distributed data. GEE models are also suitable for dealing with missing data; it is not required to exclude participants with missing questionnaires, nor should missing questionnaires be imputed beforehand. A more detailed description about GEE models is described in detail elsewhere [28,29]. We used the GEE models to analyze the association between positive and negative affect on the one hand, and momentary burden as a result of the measurement itself on the other. The variables diagnosis group, gender, age, weekly working hours, and educational level were separately added to the GEE models as independent variables to check for possible confounding. Next, the variables were added as interaction terms to the model to check for possible effect modification. A GEE model was also used to analyze the stability of the reported levels of momentary burden across the EMA questionnaires. To see whether levels of burden changed as the study period progressed, we added the variable time to the unadjusted model. All analyses were carried out using SPSS (version 25.0) and two-sides *p* values < 0.05 were considered significant (*p* < 0.017 after Bonferroni correction).

## 3. Results

### 3.1. Sample Characteristics

Table 1 shows the demographic and clinical characteristics that were assessed across the three diagnosis groups. In the overall sample, 67.0% (238 out of 384) of the participants were female, the median age was 51.0 years (IQR = 38.00–61.00), the average of weekly working hours was 20 (IQR = 0.00–35.5), and most individuals had intermediate (50.3% [193 out of 384]) or high (46.4% [178 out of 384]) education. In general, a considerable number of persons with current affective disorder and remitted persons suffered multiple affective disorder at the time of the EMA questionnaires or in their history, respectively. In total, 39.0% (39 out of 100) of the persons with current affective disorders suffered from more than one affective disorder (range 2–7) at the time of the EMA questionnaires. In the group of persons with remitted persons, 66.3% (126 out of 190) suffered from more than one affective disorder (range 2–7), in parallel or in sequence, in their life span. 

### 3.2. Burden

#### 3.2.1. Person-Mean Momentary Burden

**Figure 2:**
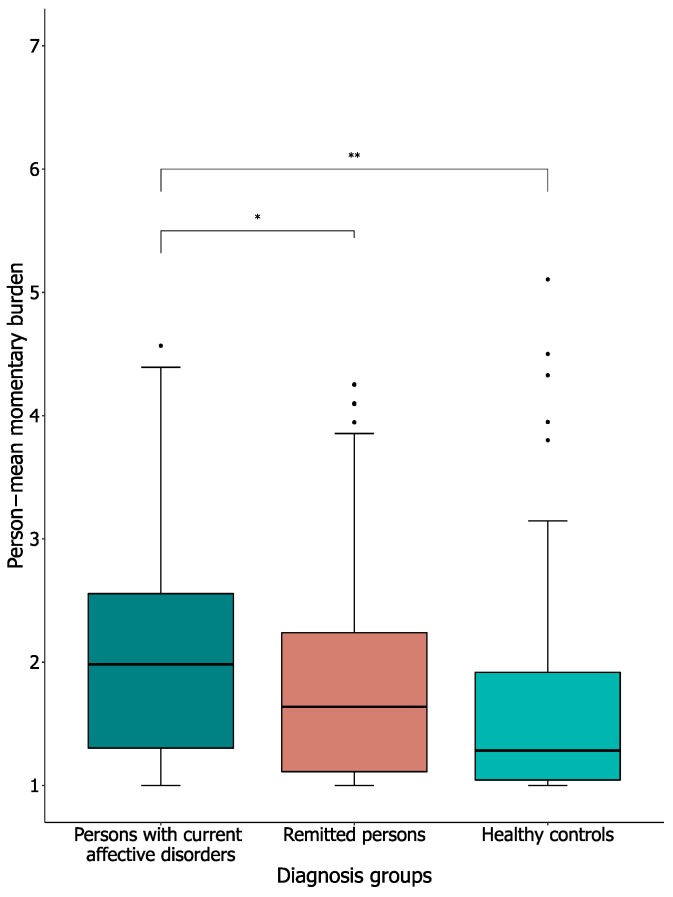
Person-mean momentary burden as reported on the EMA measures. Note: Value labels: 1 = ’No burden’; 4 = ’Moderate burden’; 7 = ’High burden’. Thick black line shows the median, error bars show the interquartile range (IQR), whiskers show +/−1.5 IQR, ● = outlier, deviates by ≥ 1.5× IQR, * = significant at *p* < 0.017 (Bonferroni-adjustment), and ** = significant at *p* < 0.0001. 3.3. Adherence to the daily EMA questionnaires.

To examine perceived burden as a result of the daily EMA questionnaires, we looked at momentary burden and at an evaluation of experienced burden over the whole two-week EMA monitoring period. Figure 2 shows the distribution of the person-means of the momentary burden in the three diagnosis groups. Tests showed a significant difference between the three groups (H[2] = 17.31, *p* < 0.0001). Subsequent, Bonferroni-corrected pairwise comparisons showed that, on average, the person-means of momentary burden reported by the persons with current affective disorders (Mdn = 1.98, IQR = 1.28–2.57) were significantly higher than in both the remitted persons (Mdn = 1.64, IQR = 1.11–2.24; U = 7229.50, *p* = 0.01) and the healthy controls (Mdn = 1.28, IQR = 1.04–1.92; U = 3098.50, *p* < 0.0001). 

#### 3.2.2. Association Between Affective States and Momentary Burden

Table 2 shows the results of the unadjusted and adjusted GEE analyses, calculated over the whole sample (with a total of 24,537 completed EMA questionnaires). First, we found a significant negative association between positive affect and momentary burden (B = −0.23, 95%CI = −0.27–0.19, *p* < 0.0001), and a significant positive association between negative affect and momentary burden (B = 0.30, 95%CI = −0.24–0.37, *p* < 0.0001). These coefficients indicate that a score of 1.00 higher on the PA scale (range 1–7) is associated with −0.23 points less reported burden (range 1–7) and 1.00 point higher on the NA scale (range 1–7) is associated with 0.30 higher reported burden (range 1–7). Hereafter, in both analyses, the variables diagnosis group, weekly working hours, gender, age, and educational background were separately added to the model to check for possible confounding. None of these variables were considered a confounder. In addition, in neither analysis did we find significant interaction between these five covariates on the one hand, and positive or negative affect on the other. Next, regards the stability of momentary burden (i.e., to see whether levels of burden changed as the study period progressed); we found that the strength of the association between reported levels of burden as a result of the questionnaires did not change significantly over time (B = 0.00, 95%CI = 0.00-0.00, *p* = 0.09). To conclude, these results indicate that lower positive affect and higher negative affect were associated with slightly higher levels of momentary burden, regardless of the presence of a current affective disorder, number of weekly working hours, age, gender, and educational background. Also, reported level of burden remained stable over time across the whole sample.

#### 3.2.3. Experienced Burden Over Whole Monitoring Period

**Figure 3:**
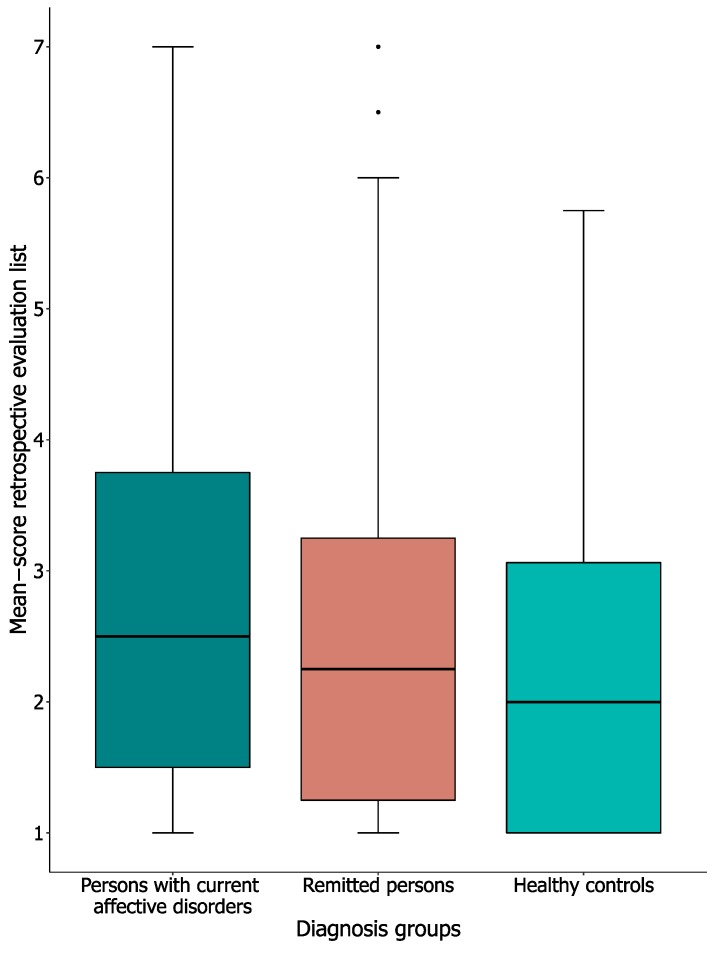
Mean scores of the retrospective evaluation of experienced burden. Note: Mean scores of the retrospective evaluation; a reflection of experienced burden over the whole EMA monitoring period. Score range between 1 and 7, higher score indicates more burden. Thick black line shows the median, error bars show the interquartile range (IQR), whiskers show +/−1.5 IQR, and ● = outlier, deviates by ≥ 1.5× IQR.

We asked the participants to evaluate the overall experienced burden of the whole two-week EMA monitoring period. Figure 3 shows the median (Mdn) and inter-quartile range (IQR) of the overall experienced burden. The persons with current affective disorders reported an average score of 2.5 (*n* = 95; IQR = 1.50–3.75), the remitted persons 2.25 (*n* = 182; IQR = 1.25–3.25), and healthy controls 2.0 (*n* = 92; IQR = 1.00–3.13). No significant inter-group differences regarding average overall experienced burden were found (H[2] = 5.56, *p* = 0.062). 

#### 3.3.1. Adherence Rates

**Figure 4:**
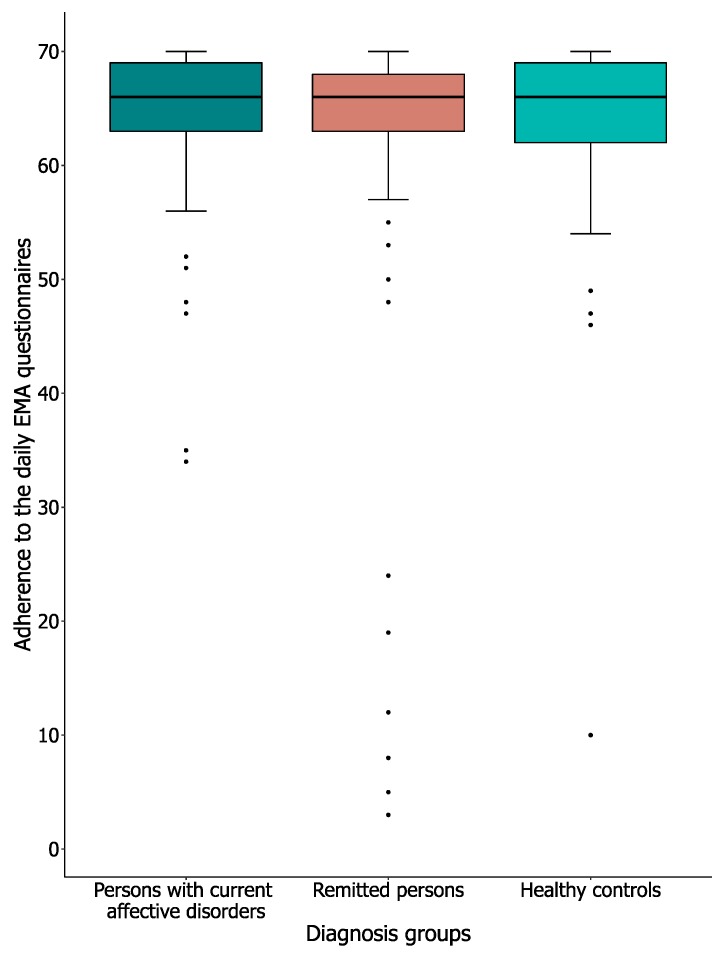
Adherence to the EMA questionnaires. Note: Participants were invited to conduct a total of 70 EMA questionnaires. Thick black line shows the median, error bars show the interquartile range (IQR), whiskers show +/−1.5 IQR, and ● = outlier, deviates by ≥ 1.5× IQR.

Adherence to the daily EMA questionnaires was our other main outcome. We looked at adherence rates, self-reported main reasons for missing questionnaires, and the number of omissions due to technical issues. Figure 4 shows the median (Mdn) and inter-quartile range (IQR) of the adherence rates to the EMA questionnaires. All groups showed the same median adherence rate (66 out of 70 [94.3%]) to the EMA measures, meaning that there is no significant difference between the persons with current affective disorders (IQR = 63.5–69.0), the remitted persons (IQR = 63.0–68.0), and the healthy controls (IQR = 62.0–69.0; (H[2] = 0.08, *p* = 0.98). These results show that adherence rates in our sample were high. 

#### 3.3.2. Reasons for Missing Daily EMA Questionnaires

After the last daily EMA questionnaire, participants were asked to list their main reasons for missing questionnaires. In total, 369 participants (out of 384) completed this addendum questionnaire-321 of which missed at least one EMA questionnaire. For 57.2% of them (184 out of 321), it appears that ‘being busy with an activity’ was one of their main reasons for missing a questionnaire. Other reasons frequently reported were ‘no network connection’ (reported by 22.4% [72 out of 321]), and ‘being asleep’ (reported by 21.2% [68 out of 321]). There are no statistically significant group differences for these and the other reasons as listed in Table 3.

Next, we checked whether EMA questionnaires were missed due to technical issues with the server. In the group of persons with current affective disorders, this was the case for 0.14% (10 out of 7000) of the questionnaires. The equivalent figure for the group of remitted persons was 0.19% (25 out of 13,300) and for the healthy controls it was 0.30% (20 out of 6,580 measures). It thus appears that, across the sample, technical issues accounted for only a small number of the missed questionnaires.

## 4. Discussion

The results of this study show that, when asked at the moment of assessing, perceived burden as a result of the ecological momentary assessment (EMA) questionnaires was slightly higher when affect was worse. Adhering to the measure was thus more burdensome for persons diagnosed with an affective disorder, than it was for remitted persons and healthy controls. Nevertheless, when asked to evaluate the overall experienced burden of the whole two-week monitoring period, no significant inter-group differences were found. Moreover, the persons with current affective disorders showed very high adherence rates regardless. 

Our findings show that when asked “in the moment”, higher negative affect and lower positive affect were associated with slightly higher levels of perceived burden as a result of the EMA questionnaires. This was the case for all diagnosis groups and regardless the number of weekly working hours, age, gender, and educational background of the participant. The persons with current affective disorders thus reported higher levels of burden by comparison to the other two groups. These findings could possibly be explained by the fact that the clinical picture of the current affective disorders involves a persistent depressed mood and/or diminished interest or pleasure in general [17,18]. Adhering to EMA questionnaires might therefore be more intrusive to persons with current affective disorders, than for persons without such a disorder. Nevertheless, across the two-week EMA monitoring period the experienced burden remained stable, and for the overall experienced burden of the whole two-week EMA inter-group differences were no longer found. 

Importantly, the higher levels of “in the moment burden” amongst the persons with current affective disorders did not result in nonadherence; on average, they completed over 94% of the EMA questionnaires—a rate on a par with the other two groups in this cohort. Moreover, the adherence rates found in this study are even higher than in previous ones with a similar population, although these studies used different EMA methods (e.g., paper-and-pencil diaries, PDAs) [8,14,15]. Previous studies using a smartphone-based method in other psychiatric populations also had lower adherence. A meta-analysis showed a pooled average adherence rate of 71% in substance abusers [30]. Note, however, the adherence rates in our study might have been influenced by the fact that participants were allowed to answer the questionnaires within 60 minutes after being prompted. Other studies often only allow for a short period of time to complete the questionnaires (e.g., up till 30 minutes) [31]. 

Our study does have some limitations, though, which should be considered when interpreting our findings. Most notably, it can be reasonably assumed that the participants in the NESDA-EMAA sample are highly motivated as they have been taking part in the NESDA study for over nine years. In addition, the results of our a relatively short EMA monitoring period might not be readily generalized to other settings that rely on longer EMA monitoring periods. Another point is that momentary burden was collected with one single item. As a result, the psychometric properties of this main outcome measure could not be assessed. In future work, it is advised to include (multiple item) questionnaires in order to report psychometric properties of the momentary burden assessment. Still, the results of the present study have important clinical implications for clinical practice. In most clinical settings, some form of real-time data is already gathered via paper-and-pencil methods (e.g., thought diaries, assessment of mood fluctuation in bipolar patients, or assessments of the frequency and intensity of panic attacks). The number of studies investigating the clinical usefulness of EMA methods via a smartphone in routine practice is also rising [32,33,34]. User experiences should also be explored in clinical settings when considering whether EMA can indeed be translated into a tool, beneficial for patients with affective disorders. 

## 5. Conclusions

In conclusion, even though the persons with current affective disorders did report experiencing more burden as a result of the EMA questionnaires when asked about this at the moment of assessment, this was not the case when asked to evaluate the whole monitoring period, and they were still able and willing to provide real-time information on their mental well-being. This study is, to the best of our knowledge, the first to systematically examine user experiences of EMA questionnaires in persons with current affective disorders while making a direct comparison with remitted persons and healthy controls in a single cohort. Although our conclusions highlight the need to examine this topic further, they do already provide important insights into the acceptability of (short-duration) EMA methods for persons with affective disorders.

## Figures and Tables

**Table 1:** Demographics of the study sample.

		Persons with Current Affective Disorder(S) Persons (*n* = 100)	Remitted Persons (*n* = 190)	Healthy Controls (*n* = 94)	Total (*n* = 384)	Test Value	*p* Value
**Demographic characteristics**							
Age		50.5 (41.5–59.0)	48.5 (36.0–61.0)	54.0 (4.0–62.0)	51.0 (38.0–61.0)	H(2) = 4.25	0.12
Gender							
	Female	64 (64.0)	132 (69.5)	51 (54.3)	238 (67.0)	χ^2^(2) = 6.35	0.04
	Male	36 (36.0)	58 (30.5)	43 (45.7)	117 (33.0)		
Educational level							
	Basic	8 (8.0)	4 (2.1)	1 (1.1)	13 (3.4)	χ^2^(4) = 14.51	0.01
	Intermediate	49 (49.0)	106 (55.8)	38 (40.4)	193 (50.3)		
	High	43 (43.0)	80 (42.1)	55 (58.5)	178 (46.4)		
Working hours (a week)		10.0 (0.0–32.0)	20.0 (0.0–32.0)	30.0 (0.0–37.0)	20.0 (0.0–35.5)	H(2) = 10.22	0.01
**DSM-IV-R Diagnosis**		**Current**	**Lifetime**				
	One more or anxiety disorder	71(71.0)	142 (74.7)				
	One or more depressive disorder	61 (61.0)	163 (85.8)				
	Generalized anxiety disorder	12 (12.0)	69 (36.3)				
	Social phobia	35 (35.0)	86 (45.3)				
	Panic disorder with agoraphobia	6 (6.0)	41 (21.6)				
	Panic disorder without agoraphobia	13 (13.0)	31 (16.3)				
	Agoraphobia	22 (22.0)	32 (16.8)				
	Major depressive disorder	57 (57.0)	160 (84.2)				
	Dysthymia	15 (15.0)	52 (27.4)				
Number of affective disorders^*^		**Current**	**Lifetime**				
	1	61 (61.0)	64 (33.7)				
	2	23 (23.0)	45 (23.7)				
	3	12 (12.0)	39 (20.5)				
	4	3 (3.0)	22 (11.6)				
	5	1 (1.0)	10 (5.3)				
	6	0 (0.0)	8 (4.2)				
	7	0 (0.0)	2 (1.1)				

Note: Data are n (%), mean (SD) or median (IQR). Kruskall–Wallis, Pearson’s chi-square and likelihood ratio tests were used as appropriate. * Affective disorders include depressive disorders (major depressive disorder, dysthymia) and anxiety disorders (social anxiety disorder, panic disorder with or without agoraphobia, agoraphobia, and generalized anxiety disorder).

**Table 2:** Association between momentary burden, affective scales and time.

		Regression Coefficient (Standard Error)	95%CI	Test Value	*p* Value
**Positive affect and momentary burden**					
Positive affect (PA) ^*^		−0.23 (0.02)	−0.27–0.19	χ^2^(1) = 130.77	<0.0001
+ diagnosis groups		−0.22 (0.02)	−0.26–0.18	χ^2^(1) = 114.36	<0.0001
+ weekly working hours		−0.23 (0.02)	−0.27–0.19	χ^2^(1) = 133.58	<0.0001
+ gender		−0.23 (0.02)	−0.27–0.19	χ^2^(1) = 131.38	<0.0001
+ age		−0.23 (0.02)	−0.27–0.19	χ^2^(1) = 120.83	<0.0001
+ educational level		−0.23 (0.02)	−0.27–0.19	χ^2^(1) = 134.67	<0.0001
					
+ PA × diagnosis groups					
	Persons with current affective disorders				
	Remitted persons ^†^	0.00 (0.05)	−0.09–0.10	χ^2^(1) = 0.01	0.92
	Healthy controls ^†^	−0.00 (0.07)	−0.13–0.13	χ^2^(1) = 0.00	0.99
+ PA × weekly working hours		0.00 (0.00)	−0.00–0.00	χ^2^(1) = 0.23	0.63
+ PA × gender ^‡^		−0.09 (0.04)	−0.17–0.00	χ^2^(1) = 3.77	0.05
+ PA × age		−0.00 (0.00)	−0.00–0.00	χ^2^(1) = 0.31	0.58
+ PA × educational level					
	Low				
	Moderate ^§^	−0.07 (0.07)	−0.22–0.08	χ^2^(1) = 0.90	0.34
	High ^§^	−0.13 (0.07)	−0.28–0.02	χ^2^(1) = 3.11	0.08
**Negative affect and momentary burden**					
Negative affect (NA) ^*^		0.30 (0.03)	0.24–0.37	χ^2^(1) = 80.53	<0.0001
+ diagnosis groups		0.29 (0.03)	0.22–0.36	χ^2^(1) = 73.09	<0.0001
+ weekly working hours		0.31 (0.03)	0.24–0.38	χ^2^(1) = 83.94	<0.0001
+ gender		0.31 (0.03)	0.24–0.37	χ^2^(1) = 81.76	<0.0001
+ age		0.30 (0.03)	0.23–0.37	χ^2^(1) = 73.79	<0.0001
+ educational level		0.32 (0.03)	0.25–0.38	χ^2^(1) = 90.22	<0.0001
					
+ NA × diagnosis groups					
	Persons with current affective disorders				
	Remitted persons ^†^	0.05 (0.07)	−0.09–0.19	χ^2^(1) = 0.48	0.49
	Healthy controls ^†^	0.09 (0.13)	−0.15–0.40	χ^2^(1) = 0.54	0.46
+ NA × weekly working hours		−0.00 (0.00)	−0.01–0.00	χ^2^(1) = 1.09	0.30
+ NA × gender ^‡^		0.04 (0.07)	−0.10–0.18	χ^2^(1) = 0.33	0.57
+ NA × age		0.01 (0.00)	−0.00–0.01	χ^2^(1) = 3.77	0.05
+ NA × educational level					
	Low				
	Moderate ^§^	0.15 (0.14)	−0.12–0.42	χ^2^(1) = 1.14	0.29
	High ^§^	0.21 (0.14)	−0.06–0.49	χ^2^(1) = 2.28	0.13
**Stability of momentary burden**					
Time^●^		0.00 (0.00)	0.00–0.00	χ^2^(1) = 2.83	0.09

Note: Generalized estimated equation models. Models are calculated over the whole sample, with a total of 24,537 completed EMA questionnaires. ^*^ Shows the unadjusted models. PA = positive affect, NA = negative affect. If appropriate, covariates diagnosis groups, weekly working hours, gender, age, educational level were separately added to unadjusted models. Hereafter, interaction terms were added to the unadjusted model. For the interaction terms, the regression coefficient of the interaction term is shown. ^†^ Persons with current affective disorders used as a reference group, ^‡^ men are used as reference group, and ^§^ low educational level is used as a reference group. ● Stability of momentary burden was measured by adding time to the unadjusted model.

**Table 3:** Self-reported main reasons for missing EMA questionnaires.

	Persons with Current Affective Disorders (*n* = 77)	Remitted Persons (*n* = 156)	Healthy Controls (*n* = 75)	Total (*n* = 321)	Test Value	*p* Value
Amount of reasons	2 (1–2)	2 (1–2)	1 (1–2)	2 (1-3)	H(2) = 3.10	0.21
**Main reasons for missing questionnaires**						
Being busy with an activity	52 (67.5)	86 (55.1)	46 (61.3)	184 (57.3)	χ^2^(2) = 3.40	0.18
No network connection	18 (23.4)	38 (24.4)	16 (21.3)	72 (22.4)	χ^2^(2) = 0.26	0.88
Being asleep	22 (28.6)	36 (23.1)	10 (13.3)	68 (21.2)	χ^2^(2) = 5.31	0.07
Technical problems	14 (18.2)	29 (18.6)	15 (20.0)	58 (18.1)	χ^2^(2) = 0.94	0.95
Did not hear the smartphone	17 (22.1)	24 (15.4)	14 (18.7)	55 (17.1)	χ^2^(2) = 1.62	0.45
Did not bring the smartphone	12 (15.6)	26 (16.7)	7 (9.3)	45 (14.0)	χ^2^(2) = 2.26	0.32
Could not make themselves do it	1 (1.3)	0 (0.0)	0 (0.0)	1 (0.3)	χ^2^(2) = 3.01	0.22
Other reason	15 (19.5)	40 (25.6)	18 (24.0)	73 (22.7)	χ^2^(2) = 1.09	0.58

Note: Data are *n* (%) or median (IQR). Only individuals who missed at least one EMA questionnaire and completed the addendum questionnaire were taken into account in this table. Participants were allowed to select more than one option. Likelihood ratio or Pearson’s chi-square tests were used as appropriate.

## References

[1] Houben M., van den Noortgate W., Kuppens P. (2015). The Relation between Short-Term Emotion Dynamics and Psychological Well-Being: A Meta-Analysis. Psychol. Bull..

[2] Lamers L., Swendsen J., Cui L., Husky M., Johns J., Zipunnikov V., Merikangas K.R. (2018). Mood Reactivity and Affective Dynamics in Mood and Anxiety Disorders. J. Abnorm. Psychol..

[3] Nelson J., Klumparendt A., Doebler P., Ehring T. (2018). Everyday Emotional Dynamics in Major Depression. Emotion.

[4] Thompson R.J., Mata J., Jaeggi S.M., Buschkuehl M., Jonides J., Gotlib I.H. (2012). The everyday emotional experience of adults with major depressive disorder: Examining emotional instability, inertia, and reactivity. J. Abnorm. Psychol..

[5] Marzano L., Bardill A., Fields B., Herd K., Veale D., Grey N., Moran P. (2015). The Application of MHealth to Mental Health: Opportunities and Challenges. Lancet Psychiatry.

[6] Myin-Germeys I., Kasanova Z., Vaessen T., Vachon H., Kirtley O., Viechtbauer W., Reininghaus U. (2018). Experience Sampling Methodology in Mental Health Research: New Insights and Technical Developments. World Psychiatry.

[7] Trull T.J., Ebner-Priemer U.W. (2009). Using Experience Sampling Methods/Ecological Momentary Assessment (ESM/EMA) in Clinical Assessment and Clinical Research: Introduction to the Special Section. Psychol. Assess..

[8] Wenze S.J., Miller I.W. (2010). Use of Ecological Momentary Assessment in Mood Disorders Research. Clin. Psychol. Rev..

[9] Shiffman S., Stone A.A., Hufford M.R. (2008). Ecological Momentary Assessment. Ann. Rev. Clin. Psychol..

[10] Ben-Zeev D., Young M.A., Madsen J.W. (2009). Retrospective Recall of Affect in Clinically Depressed Individuals and Controls. Cogn. Emot..

[11] Gloster A.T., Miché M., Wersebe H., Mikoteit T., Hoyer J., Imboden C., Bader K., Meyer A.H., Hatzinger M., Lieb R. (2017). Daily Fluctuation of Emotions and Memories Thereof: Design and Methods of an Experience Sampling Study of Major Depression, Social Phobia, and Controls. Int. J. Methods Psychiatr. Res..

[12] Myin-Germeys I., Oorschot M., Collip D., Lataster J., Delespaul P., van Os J. (2009). Experience Sampling Research in Psychopathology: Opening the Black Box of Daily Life. Psychol. Med..

[13] Armey M.F., Schatten H.T., Haradhvala N., Miller I.W. (2015). Ecological momentary assessment (EMA) of depression-related phenomena. Curr. Opin. Psychol..

[14] Aan het Rot M., Hogenelst K., Schoevers R.A. (2012). Mood Disorders in Everyday Life: A Systematic Review of Experience Sampling and Ecological Momentary Assessment Studies. Clin. Psychol. Rev..

[15] Colombo D., Fernández-Álvarez J., Patané A., Semonella M., Kwiatkowska M., García-Palacios A., Cipresso P., Riva G., Botella C. (2019). Current State and Future Directions of Technology-Based Ecological Momentary Assessment and Intervention for Major Depressive Disorder: A Systematic Review. J. Clin. Med..

[16] Walz L.C., Nauta M.H., Aan Het Rot M. (2014). Experience Sampling and Ecological Momentary Assessment for Studying the Daily Lives of Patients with Anxiety Disorders: A Systematic Review. J. Anxiety Disord..

[17] American Psychiatric Association (2013). Diagnostic and Statistical Manual of Mental Disorders.

[18] Beck A.T., Alford B.A. (2009). Depression: Causes and Treatment.

[19] Difrancesco S., Lamers F., Riese H., Merikangas K.R., Beekman A.T.F., Hemert A.M., Schoevers R.A., Penninx B.W.J.H. (2019). Sleep, Circadian Rhythm, and Physical Activity Patterns in Depressive and Anxiety Disorders: A 2-week Ambulatory Assessment Study. Depress. Anxiety.

[20] Schoevers R.A., Van Borkulo C.D., Lamers F., Servaas M.N., Bastiaansen J.A.C.J., Beekman A.T., Van Hemert A.M., Smit J.H., Penninx B.W.J.H., Riese H. Momentary affect fluctuations differ between patients with current depression and anxiety disorders, remitters and healthy controls. Psychol. Med..

[21] Penninx B.W.J.H., Beekman A.T., Smit J.H., Zitman F.G., Nolen W.A., Spinhoven P., Cuijpers P., De Jong P.J., Van Marwijk H.W., Assendelft W.J. (2008). The Netherlands Study of Depression and Anxiety (NESDA): Rationale, Objectives and Methods. Int. J. Methods Psychiatr. Res..

[22] Sytema S., van der Krieke L., Thornicroft G., Ruggeri M., Goldberg D. (2013). Routine Outcome Monitoring: A Tool to Improve the Quality of Mental Health Care. Improving Mental Health Care: The Global Challenge.

[23] Bouwmans C., De Jong K., Timman R., Zijlstra-Vlasveld M., Van Der Feltz-Cornelis C., Tan S.S., Hakkaart-Van Roijen L. (2013). Feasibility, reliability and validity of a questionnaire on healthcare consumption and productivity loss in patients with a psychiatric disorder (TiC-P). BMC Health Serv. Res..

[24] American Psychiatric Association (1994). Diagnostic and Statistical Manual of Mental Disorders.

[25] WHO (1991). Composite International Diagnostic Interview.

[26] Wittchen H.U., Robins L.N., Cottler L.B., Sartorius N., Burke J.D., Regier D. (1991). Cross-Cultural Feasibility, Reliability and Sources of Variance of the Composite International Diagnostic Interview (CIDI). Br. J. Psychiatr..

[27] Bennik E. (2015). Every Dark Cloud Has a Colored Lining: The Relation between Positive and Negative Affect and Reactivity to Positive and Negative Events. Ph.D. Thesis.

[28] Lu B., Preisser J.S., Qaqish B.F., Suchindran C., Bangdiwala S.I., Wolfson M. (2007). A comparison of two bias-corrected covariance estimators for generalized estimating equations. Biometrics.

[29] Twisk J., de Vente W. (2002). Attrition in Longitudinal Studies: How to Deal with Missing Data. J. Clin. Epidemiol..

[30] Jones A., Remmerswaal D., Verveer I., Robinson E., Franken I.H.A., Wen C.K.F., Field M. (2018). Compliance with Ecological Momentary Assessment Protocols in Substance Users: A Meta-Analysis. Addiction.

[31] Janssens K.A.M., Bos E.H., Rosmalen J.G.M., Wichers M.C., Riese H. (2018). A Qualitative Approach to Guide Choices for Designing a Diary Study. BMC Med. Res. Methodol..

[32] Bos F.M., Snippe E., Bruggeman R., Wichers M., van der Krieke L. (2019). Insights of patients and clinicians on the promise of the experience sampling method for psychiatric care. Psychiatr. Serv..

[33] Loo Gee B., Griffiths K.M., Gulliver A. (2016). Effectiveness of Mobile Technologies Delivering Ecological Momentary Interventions for Stress and Anxiety: A Systematic Review. J. Am. Med. Inform. Assoc..

[34] Schueller S.M., Aguilera A., Mohr D.C. (2017). Ecological Momentary Interventions for Depression and Anxiety. Depress. Anxiety.

